# Spatial dynamics of a zoonotic orthohantavirus disease through heterogenous data on rodents, rodent infections, and human disease

**DOI:** 10.1038/s41598-019-38802-5

**Published:** 2019-02-20

**Authors:** Sophie O. Vanwambeke, Caroline B. Zeimes, Stephan Drewes, Rainer G. Ulrich, Daniela Reil, Jens Jacob

**Affiliations:** 1 Georges Lemaître centre for Earth and Climate research, Earth & Life Institute, Université catholique de Louvain, Place Pasteur 3 L4.03.08, 1348 Louvain-la-Neuve, Belgium; 2 Friedrich-Loeffler-Institut, Federal Research Institute for Animal Health, Institute of Novel and Emerging Infectious Diseases, Südufer 10, D-17493 Greifswald-Insel Riems, Germany; 3 German Center for Infection Research (DZIF), partner site Hamburg-Luebeck-Borstel-Insel Riems, D-17493 Greifswald-Insel Riems, Germany; 4 Julius Kühn-Institute, Federal Research Centre for Cultivated Plants, Institute for Plant Protection in Horticulture and Forests, Vertebrate Research, Toppheideweg 88, D-48161 Münster, Germany

## Abstract

Zoonotic diseases are challenging to study from the ecological point of view as, broadly speaking, datasets tend to be either detailed on a small spatial extent, or coarse on a large spatial extent. Also, there are many ways to assess zoonotic disease transmission systems, from pathogens to hosts to humans. We explore the complementarity of datasets considering the pathogen in its host, the host and human cases in the context of *Puumala orthohantavirus* infection in Germany. We selected relevant environmental predictors using a conceptual framework based on resource-based habitats. This framework assesses the functions, and associated environmental resources of the pathogen and associated host. A resource-based habitat framework supports variable selection and result interpretation. Multiplying ‘keyholes’ to view a zoonotic disease transmission system is valuable, but requires a strong conceptual framework to select and interpret environmental explanatory variables. This study highlights the usefulness of a structured, ecology-based approach to study drivers of zoonotic diseases at the level of virus, host, and human - not only for PUUV but also for other zoonotic pathogens. Our results show that human disease cases are best explained by a combination of variables related to zoonotic pathogen circulation and human exposure.

## Introduction

As zoonotic pathogen circulation often relies on the interaction of susceptible living organisms and on diverse environmental parameters^1,2^, obtaining ecologically relevant insights in general epidemiological patterns and dynamics of zoonotic disease ecology is resource-intensive. On the one hand, detailed field studies often only provide a partial insight in these complex systems as they mostly focus on a small section of the system. Data documenting the circulation of a pathogen directly in the wild, in its various host species, or in all relevant habitats, are often limited temporally, spatially, or both. On the other hand, some aspects of the phenomenon are recorded spatially exhaustively, such as the human cases of a notifiable disease, but may only represent the tip of the iceberg of zoonotic pathogen circulation^3^. In this context, a robust approach for including data on the spatial and environmental determinants of zoonotic pathogen circulation is of prime importance. In this study, we investigated the contribution of three datasets, examined through a common conceptual framework, to the understanding of the ecology of orthohantavirus in Germany.

Orthohantaviruses are emerging zoonotic pathogens^4^. In Europe, *Puumala orthohantavirus* (PUUV) circulates typically in bank voles (*Myodes glareolus*)^5,6^ that live in forests and other habitats, where trees or hedges are available. Other rodent-associated human-pathogenic orthohantaviruses present in Europe are Dobrava-Belgrade, Tula and Seoul orthohantavirus, with Tula orthohantavirus infections being only rarely found in humans^7–12^. In addition, shrew-, mole- and bat-borne orthohantaviruses have been detected in Europe, but it remains unclear whether they are pathogenic to humans^13–16^. PUUV, the pathogen of focus of this study, is transmitted between individuals of a single reservoir host species through direct or indirect transmission of virus present in the habitat. Humans are accidental hosts that become infected principally by inhaling infectious aerosols lifted from places where infectious bank voles excreted urine, faeces or saliva^17^, or, in rare cases, by bites of infected reservoirs^18^. PUUV is the most common orthohantavirus species in Europe^19^ and causes the largest number of human cases. In humans, PUUV usually causes a mild to moderate form of haemorrhagic fever with renal syndrome^20^, called nephropathia epidemica (NE)^21^. Known risk factors include: living within 100 m of a forest, entering unoccupied rooms and buildings that are not well ventilated, noticing voles or vole excreta, cutting or handling firewood, and being a forestry or construction worker or muskrat hunter^22–24^.

In Germany, orthohantavirus disease cases became notifiable in 2001 and several PUUV outbreaks have been reported from various areas^6,25,26^. The understanding of spatial and temporal dynamics related to environmental determinants remains patchy, because the resolution and extent of the studies vary substantially. Local, spatially detailed studies (e.g.^27^) and others at larger extent but coarser resolution (e.g.^28^) have been carried out. Studies at a local scale identified tree seed production and especially seed mast of beech trees (*Fagus sylvatica*) as environmental determinant for human PUUV infections in Baden-Wuerttemberg and Western Thuringia empirically^28–30^ or putatively^31–33^. Studies at the federal state level in Germany in 2001–2007 showed that climatic conditions associated with high human PUUV incidences are mild winters and springs^28^ and that bank vole density is indirectly linked to weather conditions two years before, reflecting the importance of tree mast preceding a bank vole population increase^34–36^. At the level of Germany, elevated bank vole abundance due to beech mast is a driver for human PUUV prevalence^37,38^ and can be used for prediction of human infection risk^38^. The circulation of PUUV within the rodent host population is dependent on multi-annual as well as seasonal dynamics of population abundance^37^. In addition, human risk for PUUV infections is likely driven by exposure patterns. Rural dwellers were identified as more at risk than urban dwellers^39^, but humans have also been infected during recreational activities in a forested city park in Cologne^25^. Orthohantavirus circulation and disease incidence thus represents an ideal system to look into the contributions of heterogenous datasets, covering host ecology, host infection, and human exposure, to further our understanding of the ecology of zoonotic pathogens.

While there has been considerable progress in studying the ecology of PUUV^40^, a number of important questions remain. Spatial discontinuities in the distribution of infected rodents have been highlighted, such as the mismatch between the distribution of bank voles and the virus^10^ that appears to be related to the postglacial recolonization of Europe by bank voles from different refugia^30^. It is also unclear how the distribution of human clinical cases can inform about zoonotic circulation. This relates to a degree of ambiguity as to how the environmental variables may affect the system, as they may affect the virus, the rodent host, or the accidental host (humans) - directly or indirectly. For instance, forest habitat clearly attracts humans for spare time or professional activities, but also provides important resources for the hosts (e.g.^41,42^). A more systematic study using diverse data sources and a robust conceptual framework for the role of the environment can help to clarify these issues.

In this paper, we adapted a conceptual framework that takes a bottom-up approach to the ecology of PUUV. This framework considers the pathogen as the primary focus, examining potential drivers of pathogen transmission between rodent hosts and humans considering functional habitat in detail. Such an approach is common in studying aspects in conservation ecology^43,44^, as it corresponds to the classic Hutchinson’s niche concept and has been recently adapted to vector-borne diseases^45,46^. It was brought forward as a promising path to better understand the association between environment and vector-borne diseases. No single dataset available to us for Germany allows jointly investigating all environmental resources necessary for PUUV transmission. This is particularly true when considering risk to humans infections, which is the combination of hazard, that is, presence/abundance of the virus, but also human exposure to it. In this study, we used the proposed framework to jointly examine three datasets that each inform on a single aspect of the system: bank vole presence or absence, PUUV infection in bank voles, and human orthohantavirus disease case records. Each dataset offers different insights in potential environmental drivers of the spatial distribution of PUUV infection in rodent hosts and humans. A comprehensive understanding of the relevant drivers at the level of virus, host and human may help to identify features to predict human disease risk not only for PUUV but also for other rodent-borne pathogens.

## Results

### A resource-based habitat concept for PUUV

**Figure 1:**
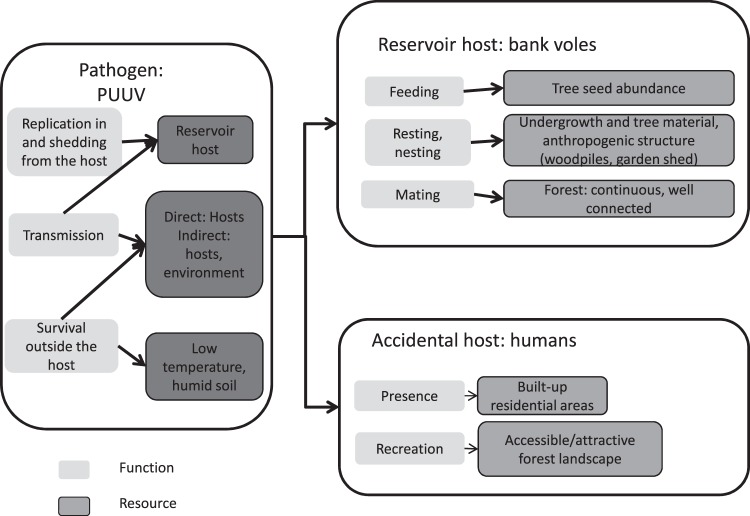
Resource-based habitat concept adapted to *Puumala orthohantavirus* (PUUV).

The resource-based habitat concept (RBHC) can be used to identify ecological resources in a systematic way, here with a focus on a pathogen and with the objective to understand the ecological requirement of its transmission^45^. We adopted the perspective of the pathogen rather than an approach focusing on habitat characteristics as the latter is often restricted to certain vegetation types. We included here an additional, non-essential resource (for the pathogen): humans, as we wanted to assess the risk for humans as well. We included only human functions and resources relevant for PUUV infection in our analyses (Fig. 1). As this study focused on the spatial distribution based on associated resources for PUUV (Table 1), we did not systematically assess elements affecting the temporal dynamics of transmission of this orthohantavirus.

**Table 1:** Variables associated to PUUV orthohantavirus resources and their hypothesized impact (‘+’ – positive effect; ‘−’ – negative effect)

Function: Resource	Environmental indicator	Hypothesized effect on the resource
**Virus**		
Survival outside the host: Low temperature, humid soil	Annual sum of precipitations (mm)	+
	Relative humidity (daily average, %)	+
	Number of dry days (day)	−
	Number of warm days (day)	−
	Maximum temperature in summer (°C)	−
	Minimum temperature in winter (°C)	−
	Snow depth (cm)	+
	Soil Water Index (relative unit)	+
Host: abundant bank voles. At metapopulation level, high level of connectedness	Forest contiguity index (relative unit)	+
	Nearest distance between forest patches (m)	−
**Host**		
Feeding: tree seed abundance	Growing season length (day)	+
	Minimum temperature in winter (°C)	+
	Snow depth (cm)	−
Resting and nesting: undergrowth and tree materials	Broad-leaved forest (%)	+
	Mixed forest (%)	+
	Coniferous forest (%)	−
Resting and nesting: anthropic structures such as woodpiles or garden cottage (*)	Built-up areas in forest ecotones (%)	+
Mating: continuous and well connected forests	Forest contiguity index (relative unit)	+
	Nearest distance between forest patches (m)	−
**Accidental host**		
Residence: built-up	Built-up (%)	+
	Population density in 2012 (inhabitants/km²)	+
Recreation: accessible/attractive forest landscape	Broad-leaved forest (%)	+
	Coniferous forest (%)	−
	Number of warm days (day)	+
	Maximum temperature in summer (°C)	+

#### Pathogen level

We postulated three essential functions for the pathogen: replication in the reservoir host, survival outside the host, and transmission. Transmission follows two pathways: either from host to host or from contaminated environment to host. We can therefore associate two resources to PUUV functions: reservoir hosts, the bank vole, and an environment (generally the soil) that permits virus survival by being humid and cold^17,27,47^. A high degree of contact between bank voles is assumed to favour virus transmission and is achieved by high connectedness at the metapopulation level^48–50^.

#### Reservoir host level

Three functions were identified for bank voles: feeding, resting and nesting, and mating. An essential resource for feeding, particularly in fall and winter, is tree seeds. The role of tree seed abundance on spatial and temporal dynamics of bank vole populations is well documented^34,50–54^. Resting and nesting resources are forest undergrowth and tree material, and also anthropogenic structures such as woodpiles or garden sheds^10^. As the bank vole is a forest species, mating opportunities rely on the distribution of potential mates, which is governed by the presence of continuous and well-connected forests^55^. We did not explicitly consider rodent movement, but movements can be assumed to take place^56–58^ within our basic spatial units of study (6 km² resolution).

#### Accidental host: humans

Humans are not necessary to the PUUV transmission cycle, but we aimed to understand how their interaction with infectious environments is affecting the incidence of the disease and therefore included them here. This was also appropriate as human clinical case records have been used as an important indicator of PUUV circulation to improve our understanding of environmental determinants of PUUV transmission^41^. The human functions relevant to PUUV transmission were residing and recreating in forested areas. The associated resources were built-up areas and accessible or attractive forest landscapes^42,59^. It should be noted that other species may be subject to orthohantavirus spillover, but humans are our accidental host of focus.

### Presence of infected bank voles

**Figure 2:**
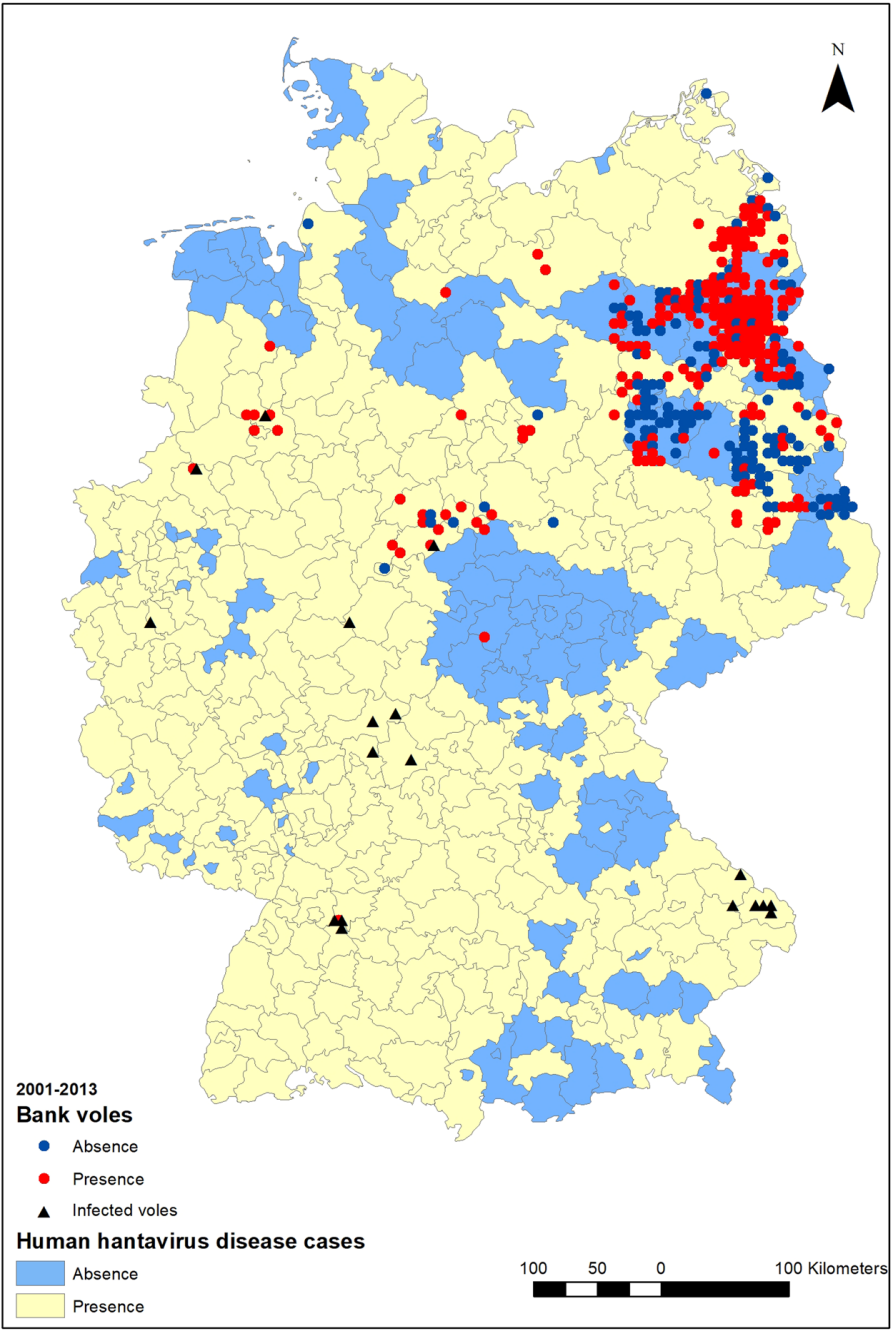
Data distribution for general bank vole presence, presence of PUUV infected bank voles and occurrence of human orthohantavirus disease cases in Germany.

Overall, several of the variables selected to measure resources were statistically significantly related to places of bank vole presence/absence but only some contrasted infection in voles vs. bank vole presence (Fig. 2, Table 2).

**Table 2:** Environmental factor values for bank vole presence vs. absence and bank vole infected vs. presence.

	Bank vole: Presence vs. absence			Bank vole: infected vs. present		
	Median		Wilcoxon test:	Median		Wilcoxon test
	Absence	Presence	n/30 results with p < 0.05	Vole	Infected	n/30 results with p < 0.05
Annual sum of precipitation (mm)	595.13	812.88	30	614.78	843.21	30
Relative humidity (%)	77,60	79.82	27	79.33	79.44	1
Number of dry days	24.31	21.64	30	23.79	21.51	30
Number of warm days	10.95	9.04	28	9.63	9.87	6
Maximum temperature in summer (°C)	23.95	22.85	30	23.15	22.87	0
Minimum temperature in winter (°C)	−2.17	−1.73	30	−1.97	−2.16	2
Snow depth (cm)	0.84	0.85	0	0.82	1.06	0
Soil Water Index (relative unit)	146.58	137.58	9	139.43	135.84	0
Forest contiguity (relative unit)	0.67	0.70	0	0.69	0.63	0
Nearest distance between forests (m)	502.93	541.61	14	518.61	524.59	0
Growing season length (days)	262.13	269.87	13	264.16	260.5	0
Broad-leaved forest (%)	1.34	7.62	28	6.29	8.33	0
Mixed forest (%)	1.19	3.40	13	2.91	16.32	30
Coniferous forest (%)	30.27	11.37	11	17.63	14.80	2
Built-up areas in forest ecotone (%)	3.07	4.47	1	0.59	0.12	0

For bank vole presence vs. absence, the annual sum of precipitation, relative humidity, number of dry days, higher temperature in summer, lower temperature in winter and broad-leaved forest were significantly different between places of presence and absence in 27 or more replications of the test, with the expected effect (as per Table 1). The differences in nearest distance between forest patches, growing season length, mixed forest and coniferous forest cover between bank vole presence and absence were significant in 11 to 21 replications of the Wilcoxon test. The directions of the differences between places of presence and places of absence were as expected for all variables but the nearest distance between forest patches. Differences in the soil water index, and built-up areas in the ecotone between bank vole presence and absence were significant only in one or two replications, and we did not interpret these results further.

For PUUV infection in bank voles, the annual sum of precipitation, the number of dry days and the proportion of mixed forest differed significantly 29 or 30 times out of 30 replications. The differences in values between the two groups were in the direction expected. Differences in relative humidity, the number of warm days, and proportion of coniferous forest were significant only in six or fewer replications of the test. No other variable was significant in any replication of the Wilcoxon test.

### Presence of human orthohantavirus disease cases

The predictive power of the Boosted Regression Tree (BRT) model for the presence of human orthohantavirus disease cases was good with an AUC of 0.93 ± 0.01 (AUC based on cross-validation = 0.68 ± 0.04).

**Figure 3:**
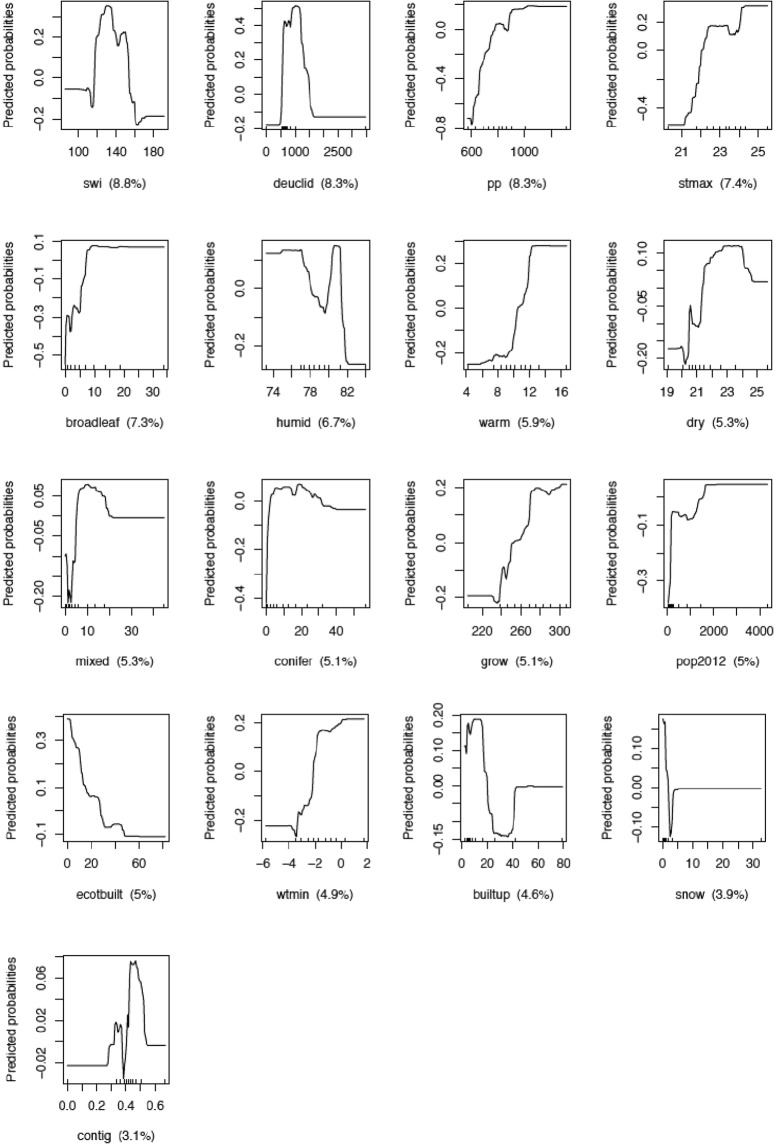
Response curves of variables according to predicted probabilities in the presence of human orthohantavirus disease cases (variables ordered by relative importance). Swi = soil water index (relative unit), deuclid = nearest distance between forest patches (m), pp = annual sum of precipitation (mm), stmax = maximum temperature in summer (°C), broadleaf = broadleaf forest (%), humid = relative humidity (%), warm = number of warm days, dry = number of dry days, mixed = mixed forest cover (%), conifer = coniferous forest cover (%), grow = length of the growing season (days), pop2012 = human population density (inhabitants/km²), ecotbuilt = built-up areas in forest ecotones (%), wtmin = minimum temperature in winter (°C), builtup = built-up areas (%), snow = snow depth (cm), contig = forest contiguity index (relative unit).

Figure 3 shows the response curves of all variables included in the model. The five variables with the highest relative importance (>7%) were soil water index, nearest distance between forest patches, precipitation, maximum temperature in summer, and broad-leaved forest cover.

Variables with a global positive effect on the probability of presence of human orthohantavirus disease cases were broad-leaved forest cover, maximum temperature in summer, precipitation, human population density, lower temperature in winter, number of warm days, and growing season length. Variables with a negative effect on the probability of presence of human orthohantavirus disease cases were relative humidity and built-up area in forest ecotone. Variables with non-linear effect were soil water index, nearest distance between forest patches, mixed forest and coniferous forest cover. Forest contiguity and snow depth (0–4 cm) had mixed effects on the probability of the presence of human orthohantavirus disease cases.

## Discussion

We adapted the RBHC to the case of PUUV and used large-scale high-resolution data covering various compartments of the orthohantavirus transmission system in Germany to investigate the importance and role of environmental determinants, and how partial data can inform on these.

Variables expected to be associated with bank vole presence were found often significant in the Wilcoxon tests. This included variables describing environments favourable to voles, such as broad-leaved forests and precipitation. Some relations however were in the direction opposite to our expectation. This included the nearest distance between forest patches, where areas with more distant patches were related to presence, but areas with less distant patches were not. This was not observed in all comparisons, and may relate to an inadequacy of this particular variable as a proxy for forest connectivity at this scale. The amount of built-up area in the forest ecotone was expected to be associated to a greater availability of shelter for voles, but this may not represent a good proxy for the preferred shelter of bank voles in Germany. Vole presence was nearly always associated with broad-leaved forest and sometimes with mixed forest, probably the secondary habitat choice of voles. Coniferous forests were more abundant around absence sites, but only some tests were significant.

Virus presence in bank voles was associated with fewer variables than vole presence/absence, mostly with variables hypothesised to relate to the virus functions and resources, but not always with the expected sign. This was the case for relative humidity and the number of warm days. However, with only one and six Wilcoxon tests significant (p < 0.05), respectively, we did not consider this significant. Virus presence was, however, always associated with higher percentages of mixed forests, consistent with^29^. This may point to environmental influences on vole responses to infection and genetic polymorphism^60^. Variables we had hypothesised to associate to resources of the virus also predicted vole presence (annual sum of precipitation, relative humidity, number of dry days, number of warm days, warmer summer). This could relate to more favourable conditions for vole food supply. A multivariate model examining the joint effect of potential predictors of the function ‘food’ could allow disentangling the role of various resources and their proxies and should be considered in further work. Previous studies have emphasised the predictive role of food availability to voles through tree seed mast^29–31,39,54^, but also the need to consider forest more broadly than as purveyor of seeds^61^. Interpreting these results should however be done with care as the status of the voles in the presence dataset is unknown. Poor representativity over the entire German territory should also be considered.

The main output of interest of the BRT model are the response curves allowing us to examine the effect of environmental proxies on the probability of human orthohantavirus infections at the county level (Fig. 3). In this model, additional variables relating to human exposure were included and some variables examined for bank vole presence need to be interpreted also as proxies for the attractiveness of landscape, which can be coherent with bank vole resources (e.g. broad-leaved forests) or not (e.g. number of warm days). The results point to rural areas as being more at risk, as per the negative relationship with the percentage of built-up area in a landscape – even though there is a positive association with human population density. This may suggest that, while areas more rural (less urbanized), are more suitable to infected voles, human disease cases may only be recorded in those areas with sufficient human exposure (human population). Most results were consistent with the general bank vole presence and infected bank vole analyses. Warmer temperatures in summer were not consistent with bank vole presence or infection analyses, but were consistent with the hypothesised effect on landscape attractiveness for humans. A similar analysis can be conducted for the number of dry days, even though we had not hypothesised an effect on human cases for this variable. While human behaviour with regard to weather conditions, in terms of outdoors activities, particularly related to recreation, would deserve to be studied with temporal detail, this highlights the fact that when a zoonotic disease is studied through the’keyhole’ of human cases, such possible contradictory effects must be considered. Also worth mentioning is the comparable relative importance of all variables included (Fig. 3). No group of variables, related to virus, host or human, stands out to be particularly important. This is coherent with other results of ^62^, who obtained higher R² values for regression models looking at the effect of environmental variables focused on vole ecology fitted on vole records, than when looking at NE case records.

Results for human cases were partly in accordance with^41^, with similar response curves for broad-leaved forests, mixed forests, and coniferous forests as well as precipitation, as compared for the Western European broadleaf forests ecoregion and the Temperate Northern Atlantic ecoregion, which cover most of Germany and were also studied there. However, other variable responses differed, but that study did not cover all ecoregions encountered in Germany and used a somewhat different set of variables.

Datasets documenting various aspects of the ‘zoonotic pathogen circulation iceberg’^3^ offer the opportunity to test various hypotheses relating to the effect of environment on three levels: the virus, the host, and the human accidental host. Because each of these levels is susceptible to environmental conditions, being able to examine them individually is valuable. Our hypotheses as well as our results suggest that the effect of an environmental factor can work in different directions depending on the level considered and that effects on all levels need to be kept in mind when examining the visible tip of the ‘zoonotic iceberg’ – often, human case records. This is consistent with issues raised by^41^ concerning various scenarios for the associations between a pathogen’s presence and environmental factors. In our case, the data related to the pathogen and the host could be considered as’keyholes’ in the sense that they offer only partial views because of the data’s spatial and temporal resolution, but each contributes relevant information.

The widely differing extent and resolution of the data was the principal challenge of utilising these datasets jointly, somewhat limiting the detail and comparability of methodologies chosen. While practical constraints (e.g. resources needed for host sampling) and other issues limiting data resolution (e.g. confidentiality issues for human health-related data, bank vole sampling focused on endemic regions and few areas where PUUV has not been recorded) cannot be altogether easily lifted, the possibility to cross-use such datasets should be more carefully considered when designing data collection and analysis protocols. Spatial bias in sampling, as can be observed for the bank vole data, if considering the entire German territory, should in particular be avoided, and a sampling accounting for the distribution of environmental variables of interest, should be considered^63^. Records of absences, though most challenging to collect, also bear important information. The use of human case records poses a pervasive challenge because infection may be acquired in other places than the place of residence but the latter is used to record cases. However, the obvious association of human PUUV cases with rural areas we identified and strong correlations of human data and environmental parameters found in previous studies^50,54^ suggest that such uncertainties may be limited regarding general PUUV ecology. However, the potential discrepancy between place of infection and place of record may matter for fine-scale studies of human infection risk and spread of rodent-borne pathogens in the landscape. Therefore, recording the place of infection should be encouraged, but we acknowledge the practical difficulties associated. The reliance on serological tests, which generally does not allow to identify different orthohantaviruses furthers these uncertainties^64^.

Our RBHC concept helped to structure hypotheses for the various elements of the transmission system: virus, host, and human. We examined each with respect to their functions and associated environmental resources. We selected environmental data as proxies to these resources. In the plethora of environmental data available, RBHC helps selecting and interpreting environmental proxies more specifically with greater attention to the biological meanings. Such a structured approach to the ecology of the system is largely absent in many studies on the effect of the environment on zoonotic diseases^45,65^, which may not be a problem as such for predictive purposes. However, it is needed for the understanding of processes that regulate pathogen epidemiology and for environmental modelling to make a meaningful contribution to disease and risk management. This is particularly true when using human disease case records, often the most spatially exhaustive source, to investigate the ecology of vector or hosts. Our BRT results suggest, in parallel to other results^62^, that human exposure needs to be accounted for when considering PUUV in humans.

Still, the RBHC proposed here for PUUV transmission could be improved further by including temporally dynamic variables known to affect for example vole population dynamics, such as the variability of food availability via tree seed mast^29,50^ and winter survival. Stating the scale of work more explicitly would also allow improvement as the importance and effect of environmental variables may differ whether the broad scale is considered (country scale) or the fine scale (regional scale where bank voles were sampled more intensively).

In a context of better availability of environmental data, as well as pathogen related data, a fine tuned, ecologically meaningful selection of predictors as allowed using the RBHC, and thorough understanding of what each’keyhole’ dataset allows to study is of great interest. Further developments with interesting potential would be to diversify the scale of studies to investigate the effect of variables such as forest structure. Refining current practices of data collection, e.g. including information about the place of infection for humans, is of great value for the assessment of environmental determinants of human infection risk. Additional’keyholes’ could be considered. In the case of orthohantavirus transmission this could be an environment compartment where the virus is known to survive for extended periods of time. An obvious and likely necessary development of the RBHC would be the inclusion of relevant temporal dynamics, because, in the case of PUUV, there are temporal patterns in vole population as well as disease risk dynamics.

## Conclusion

Sets of data pertaining to various elements of PUUV circulation in Germany were compared using a common set of environmental variables selected based on a RBHC. Our results highlight the usefulness of a structured, ecology-based approach to formulation of hypotheses and modelling, in the PUUV system as well as for other systems. It also highlights that results pertaining to studies of diverse indicators are difficult to compare in relation to the different roles environmental resources can play depending on the level (virus, host, human) considered. Adopting a conceptual framework articulated around the ecology of the system rather than around data availability will help in furthering the field of disease ecology in general and to unify sometimes disparate results achieved in studies of particular elements of the pathogen-host-human complex.

## Methods

### Data

#### Presence/absence of bank voles

Bank vole abundance data in 2001–2013 were available from forest authorities for eight federal states of Germany: Lower Saxony (LS) for 2001–2010, Hesse (HE) for 2006–2010, Saxony-Anhalt (SA) for 2001–2006 (Northwest German Forest Research Station and the Lower Saxony State Office for Consumer Protection and Food Safety), Brandenburg (BB) for 2001–2011 (Brandenburg Forestry State Agency), Mecklenburg-Western Pomerania (MW) for 2001–2006 (Mecklenburg-Western Pomerania National Forest), Thuringia (TH) for 2001–2010 (ThüringenForst), Baden-Wuerttemberg (BW) for 2010–2013 and North Rhine-Westphalia (NW) for 2010–2013 (Julius Kühn-Institute, Institute for Plant Protection in Horticulture and Forests, Vertebrate Research). Abundance data were the number of individuals per 100 trap nights estimated from the standard snap trapping method of forestry authorities in Germany (for details see^34^). Either 50 traps were set along one or more transects for two nights or 100 traps for one night. As some trapping locations were in close proximity to each other, coordinate points were aggregated on a grid of 6 km² resolution. If the bank vole abundance in a cell was ≥1, the centre of the cell was considered as a bank vole presence record. If no bank vole occurred, it was considered as an absence point (Fig. 2). The majority of data points originated from BB and MW, totalling 335 points (223 presences) after aggregation. Only 35 sampling sites (8 absences and 27 presences) were located outside BB and MW, which implies that these two regions are overrepresented. They are also regions where no human cases of PUUV were recorded due to the absence of PUUV in bank vole populations^30^. To balance for overrepresentation of these environments and absences, 35 sites (with 27 absences and 8 presences) were randomly selected, with 30 repetitions, from BB and MW and used for analyses, rather than the entire dataset.

#### Presence of infected bank voles

Coordinates of records of PUUV infected bank voles were extracted from the data published by^66^, seven sites sampled in 2004^25^, one site sampled in 2005^26^, and 19 sites sampled in 2010. Samples were tested serologically and genetically for PUUV infection. PUUV positive points were also aggregated on a grid of 6 km² cells (Fig. 2). After aggregation, 18 presence points remained. The 18 points of presence of infected voles were compared to 54 points of bank vole presence from the bank vole presence/absence data (27 from BB and MW and 27 from the rest of the country). This was repeated 30 times with a new random selection of 27 presences from BB and MW. We adopted this strategy to account for the spatial distribution of samples, which is unbalanced across environmental gradients throughout Germany. In doing so, we followed results^64^ that indicate that better modelling accuracy is reached by a stratified sampling than by a random one.

#### Human case records

Human orthohantavirus disease cases were reported to the Public Health Institute (Robert Koch-Institute, RKI) by county from 2001 to 2013 (https://survstat.rki.de/, data status: 16.10.2013). If there was at least one case recorded in the county during this period, the county was considered as a presence of human orthohantavirus disease cases (Fig. 2). If there was no record, the county was considered as an absence for human orthohantavirus disease cases.

#### Environmental data

Environmental variables were selected using the RBHC framework. Table 1 summarizes the environmental indicators identified and their hypothesized effects on the resources. They were calculated and averaged at the county level for the human orthohantavirus disease case dataset and averaged for an area of 3-km diameter around the points (ArcGIS 10.1) for the bank vole and infected vole datasets.

Climatic variables were extrapolated by kriging from meteorological stations (ECA&D, European Climate Assessment & Dataset). On average, 601 stations recorded the variables identified in Table 1 from 2001 to 2013. The kriging was done per variable per year with a raster resolution of 1 km using the kriging function with a spherical semi-variogram (ArcGIS 10) and then averaged by raster cell over the period covered. The percentage of land use classes was calculated for the counties (human orthohantavirus disease case dataset) and for the 3 km buffers (bank voles and infected voles datasets) for coniferous forests, broad-leaved forests, mixed forests and built-up areas (2006 CORINE Land Cover, EEA, www.eea.europa.eu/data-and-maps/data/corine-land-cover-2006-raster-3). The proportion of built-up areas in an ecotone of 250 m around forests was also calculated. Forest contiguity and the minimum Euclidian distance between two forest patches were included to represent forest connectivity (calculated with FRAGSTATS, version 4, http://www.umass.edu/landeco/research/fragstats/fragstats.html). The population density in 2012 per municipality (derived from the population in 2012, Statistisches Bundesamt, https://www.destatis.de) was added to the human orthohantavirus disease case dataset to reflect human distribution.

### Statistical analyses

The heterogeneous spatial distribution of the data pertaining to bank vole presence and infection prevented the elaboration of detailed, multivariate statistical models. As we selected a data subset for a balanced spatial representation of the entire country, we limited the analyses to two-way comparisons of the environmental characteristics of bank vole and virus habitat.

### Presence of bank voles

Wilcoxon tests (R version 3.1.0) were performed to compare the environmental indicators between points of presence and absence of bank voles with the 30 repeated data samples. The median of the presence and absence samples of significant tests (p < 0.05) was averaged and the number of significant outcomes from 30 repetitions counted.

### Presence of infected bank voles

18 sites of presences of infected voles were compared to 54 sites of general presence of bank voles (infection status unknown) using Wilcoxon tests. This was also repeated 30 times, each time with a different random subsample of presences in Brandenburg and Mecklenburg-Western Pomerania. Results were summarized showing the average median of significant results and the number of significant outcomes (p < 0.05) from 30 repetitions.

### Presence of human orthohantavirus disease cases

Boosted regression trees (BRT) were used to model the presence-absence of human orthohantavirus disease cases^67^ (’gbm’ package in R). This boosted classification method allows to model non-linear responses. All variables listed in Table 1 for virus, host and humans were included in the model. The modelling parameters used were 5 for the tree complexity, 0.5 for the bag fraction, and 0.001 for the learning rate. Tree complexity refers to the degree of interactions considered by the model fitting, the bag fraction indicates the fraction of observations used in any step, and the learning rate indicates the contribution of each tree to the growing model^67^. Colinearity between predictors was tested using Kendall’s tau. If pairs of variables had a correlation over 0.6, the variable was withdrawn and the shape of the curves of predicted probabilities examined. As such tests yielded no major difference in the curve shapes, we kept a full model. The predictive power of the model was evaluated with the area under the curve (AUC)^68^. An AUC of 0.5 indicates a random model, of ≥0.7 a good model, and of 1 a perfect model. Because of spatial autocorrelation and possible over-fitting^69^, AUCs based on 10-fold cross-validation were also computed. The model outputs used were the relative importance, a weighted measure of the number of times the variable was used to build the model, and response curves, graphs with the evolution of the fitted probabilities according to the variable. Only the global trends of the response curves were considered, because local peaks may result from interactions^67^.

## Data Availability

Environmental data were drawn from publicly available sources. Bank vole data were drawn from previously published sources. Disease cases were drawn from databases at the Robert Koch-Institute.
